# Prediction of liver fibrosis severity in alcoholic liver disease by human microfibrillar‐associated protein 4

**DOI:** 10.1111/liv.14491

**Published:** 2020-05-10

**Authors:** Bjørn S. Madsen, Maja Thiele, Sönke Detlefsen, Mia D. Sørensen, Maria Kjærgaard, Linda S. Møller, Ditlev N. Rasmussen, Anders Schlosser, Uffe Holmskov, Jonel Trebicka, Grith L. Sorensen, Aleksander Krag, GALAXY consortium

**Affiliations:** ^1^ Department of Gastroenterology and Hepatology Odense University Hospital Odense C Denmark; ^2^ OPEN, Odense Patient Data Exploratory Network Odense University Hospital Odense C Denmark; ^3^ Department of Pathology Odense University Hospital Odense C Denmark; ^4^ Institute of Clinical Research University of Southern Denmark Odense C Denmark; ^5^ Institute of Molecular Medicine University of Southern Denmark Odense C Denmark; ^6^ Department of Internal Medicine University Clinic Frankfurt Frankfurt Germany; ^7^ European Foundation for the Study of Chronic Liver Failure ‐ EF Clif Barcelona Spain; ^8^ Institute for Bioengineering of Catalonia Barcelona Spain

**Keywords:** biomarker, cirrhosis, extracellular matrix protein, liver biopsy, non‐invasive testing

## Abstract

**Background:**

Alcoholic liver disease (ALD) is a public health concern that is the cause of half of all cirrhosis‐related deaths. Early detection of fibrosis, ideally in the precirrhotic stage, is a key strategy for improving ALD outcomes and for preventing progression to cirrhosis. Previous studies identified the blood‐borne marker human microfibrillar‐associated protein 4 (MFAP4) as a biomarker for detection of hepatitis C virus (HCV)‐related fibrosis. Aim: To evaluate the diagnostic accuracy of MFAP4 to detect ALD‐induced fibrosis.

**Method:**

We performed a prospective, liver biopsy‐controlled study involving 266 patients with prior or current alcohol overuse. Patients were split into a training and a validation cohort.

**Results:**

MFAP4 was present in fibrotic hepatic tissue and serum MFAP4 levels increased with fibrosis grade. The area under the receiver operating characteristic curve (AUROC) for detection of cirrhosis was 0.91 (95% CI 0.85‐0.96) in the training cohort and 0.91 (95% CI 0.79‐1.00) in the validation cohort. For detection of advanced fibrosis, the AUROC was 0.88 (95% CI 0.81‐0.94) in the training cohort and 0.92 (95% CI 0.83‐1.00) in the validation cohort. The diagnostic accuracy did not differ between MFAP4 and the enhanced liver fibrosis (ELF) test or transient elastography (TE) in an intention‐to‐diagnose analysis. MFAP4 did not predict hepatic decompensation in a time‐to‐decompensation analysis in a subgroup of patients with cirrhosis.

**Conclusion:**

MFAP4 is a novel biomarker that can detect ALD‐related fibrosis with high accuracy.

**Abbreviations**
ALD: alcoholic liver disease
AP: alkaline phosphatase
AST: aspartate transaminase
AUROC: area under the receiver operating characteristics curve
BMI: body mass index
CI: confidence interval
CRP: C‐reactive protein
ELF: enhanced liver fibrosis test
GGT: gamma‐glutamyl transferase
HCV: hepatitis C virus
HR: hazard ratio
MFAP4: microfibrillar‐associated protein 4
NAS‐CRN: non‐alcoholic fatty liver disease activity score‐clinical research network
NPV: negative predictive value
PPV: positive predictive value
TE: transient elastography

**Key points**

There is a lack of new accurate diagnostic tools to detect alcoholic liver disease in an early reversible stage. In this study, we prove that the protein MFAP4 is present in the liver and blood in patients with alcohol overuse and document that it can be used to precisely assess the severity of scar tissue in the liver.

## 1 INTRODUCTION

Mortality attributable to cirrhosis has been rising in the United States since 2009 and increasingly affects young people caused by the development of end‐stage alcoholic liver disease (ALD).^1^, ^2^ The recommended strategy to address the burden of ALD is early detection and alcohol abstinence as these actions can improve histological findings, decrease the likelihood of progression to cirrhosis and improve survival rates.^3^, ^4^, ^5^, ^6^, ^7^ Additionally, early detection of ALD enables the implementation of evidence‐based prophylaxis against liver‐related complications and reinforces disease awareness that can positively modify drinking behaviours.^8^

Unfortunately, the majority of ALD patients are diagnosed in an advanced fibrotic stage for which the prognosis is poor even if abstinence is achieved and treatment guidelines are followed.^9^ Thus, patients should ideally be identified before their disease progresses to advanced fibrosis, as this point marks the threshold for severely increased rates of liver‐ related mortality.^6^ Such early diagnosis should be achievable as nearly half of ALD patients have interacted with the healthcare system for an alcohol‐related purpose prior to developing alcoholic cirrhosis.^10^, ^11^

The current gold standard reference for determining the level of hepatic fibrosis and exclusion of co‐existing liver disease is a liver biopsy.^12^ However, because of the invasiveness and low tolerability by patients, this procedure is not suitable as a screening tool to detect early stage ALD.^13^ Recent advances in non‐invasive testing using transient elastography (TE) and serum markers, such as the enhanced liver fibrosis test (ELF), may address some of these shortcomings.^14^, ^15^ Both ELF and TE have high diagnostic accuracy for the detection of ALD‐related fibrosis, but both approaches are costly and frequently unavailable outside tertiary hospitals. Hence, there is an unfulfilled need for new biomarkers to detect fibrosis among patients with suspected ALD. Likewise, there are no tools available to identify patients who are most at risk for disease progression. Such tools would allow optimal allocation of healthcare resources and tailoring of treatment to individuals who are most in need.

The human microfibrillar‐associated protein 4 (MFAP4) is ubiquitously distributed in the extracellular matrix in the human body.^16^ Although its biological properties are not fully understood, MFAP4 is involved in orchestrating extracellular matrix remodelling during tissue repair.^17^, ^18^, ^19^, ^20^ MFAP4 was originally identified as a candidate biomarker of liver fibrosis from proteome analyses of microdissected cirrhotic septa isolated from patients infected with hepatitis C virus (HCV).^21^ Subsequently, two studies confirmed the applicability of MFAP4 for the detection of HCV‐induced liver fibrosis.^22^, ^23^ Owing to its reported diagnostic accuracy and robustness against variations in sampling handling and storage, serum MFAP4 has the potential to translate into a clinically meaningful diagnostic tool.^24^ However, the diagnostic accuracy of serum MFAP4 to detect ALD‐induced fibrosis has not been previously evaluated, nor has it been compared to that of other widely used non‐invasive techniques such as TE or the ELF test.

In this study, we aimed to evaluate whether MFAP4 is upregulated during different stages of ALD‐induced fibrosis and to compare the diagnostic accuracy of serum MFAP4 to that of TE and ELF. We also evaluated the prognostic potential of MFAP4 among patients with ALD‐induced cirrhosis.

## 2 METHODS

We performed a prospective, biopsy‐controlled, single‐centre study with an internal validation cohort. Blood samples from 50 healthy gender‐ and aged‐matched participants were used to determine the concentration of MFAP4 in healthy individuals. The studies were approved by the ethics committee of the Region of Southern Denmark (S‐20120071, S‐20160021, S‐20160006G) and adhere to the 2013 Helsinki Declaration. The studies are registered in the Odense Patient Data Exploratory Network (OPEN) under study identification numbers OP_040 (https://open.rsyd.dk/OpenProjects/da/openProject.jsp?openNo=40) and OP_239 (https://open.rsyd.dk/OpenProjects/openProject.jsp?openNo=239&lang=da). This report follows the Liver‐Fibro STARD checklist (Supporting Information).^25^ All authors had access to the study data and reviewed and approved the final manuscript.

### 2.1 Study population

This study included 266 patients with prior or current alcohol overuse, defined as more than 24 g and 36 g per day for women and men, respectively, for more than 1 year. Additional inclusion criteria were age 18‐75 years old and informed consent to undergo a liver biopsy. Patients were recruited consecutively from two municipal alcohol rehabilitation centres and from three outpatient hospital liver clinics in the Region of Southern Denmark. All participants consented after receiving verbal and written information. All patients were at significant risk for ALD that justified performance of a liver biopsy. The criteria were revised in January 2016 at which time patients younger than 30 years old and a liver stiffness below 6.0 kPa by TE were excluded based on our previous findings that patients who met these criteria did not have severe fibrosis.^26^ Exclusion criteria were decompensated liver disease with clear clinical signs of cirrhosis, severe alcoholic hepatitis (defined by clinical criteria in the form of new onset of icterus and impairment in liver function in a patient with excessive alcohol overuse), debilitating disease with an expected survival of less than 1 year, concurrent liver disease, hepatic congestion or inability to comply with the study protocol. The healthy control cohort included 50 participants. The inclusion criterion for the control cohort was age 18‐75 years old. Exclusion criteria were ongoing alcohol intake above 60 g/wk, daily alcohol intake or binge drinking habits. Individuals with prior alcohol abuse, body mass index (BMI) above 28, concurrent liver disease, comorbidity or daily intake of any medication other than mild pain relievers (over‐the‐counter non‐steroidal anti‐inflammatory drugs or paracetamol) were also excluded from the control cohort. All investigations were performed on the same day at Odense University Hospital (OUH), according to standard operating procedures after an overnight fast.

### 2.2 Histological and immunohistochemical studies

Liver biopsies were performed percutaneously with a 17‐G Menghini suction needle (Hepafix, Braun, Germany). We considered biopsies to be of adequate quality if they were > 10 mm long and contained > 5 portal tracts or if a regeneration nodule was present. A single experienced pathologist evaluated the biopsies according to the Kleiner fibrosis stage and non‐alcoholic fatty liver disease activity score (NAS‐CRN).^27^ According to the Kleiner fibrosis stage, F0 is no fibrosis, F1 is perisinusoidal or periportal fibrosis only, F2 is perisinusoidal fibrosis in combination with portal or periportal fibrosis, F3 is bridging fibrosis and F4 is cirrhosis. We classified ≥ F3 as advanced fibrosis. The NAS‐CRN is a semiquantitative score of steatosis (0‐3), ballooning (0‐2) and lobular inflammation (0‐3). MFAP4 immunostaining was performed at the Department of Pathology, OUH, Denmark. Liver biopsies were fixed in formalin and embedded in paraffin. We cut 4‐µm thick sections with a microtome and mounted the slices on FLEX IHC slides (Dako, Glostrup, Denmark). Sample paraffinization, epitope retrieval with protease 1 (for 8 minutes) and blocking of endogenous peroxidase activity were performed using a Discovery Ultra Immunostainer (Ventana Medical Systems, Tucson, AZ) with an OptiView‐DAB (8‐8) detection kit (Ventana Medical Systems, Tucson, AZ). A monoclonal mouse anti‐MFAP4 primary antibody was used at a dilution of 1:200 (HYB7‐14, produced by Prof. Dr Uffe Holmskov, University of Southern Denmark ^24^). Slides were washed, dehydrated and mounted with coverslips using a Tissue‐Tek Film coverslipper (Sakura, Alphen aan den Rijn, The Netherlands). A tissue microarray with different types of normal tissues, such as skin, tonsil, lung, spleen, prostate, testis, uterus, kidney, gallbladder, normal liver and normal pancreas, was used as control. The walls of vessels in these tissues were used as positive control. Hepatic expression of MFAP4 was semiquantitatively evaluated in a subgroup of 116 patients. For this study, we designed a 6‐ordered score defined as: 0: little or minimal MFAP4 expression; 1: MFAP4 expression in a few portal tracts and/or zone 3 in a few lobuli; 2: MFAP4 expression in many portal tracts and/or zone 3 in many lobuli; 3: MFAP4 expression in all portal tracts and in a few fibrotic septa; 4: MFAP4 expression in all portal tracts and in many fibrotic septa; 5: MFAP4 expression in many fibrotic septa and a few regenerative nodules; 6: MFAP4 expression in many fibrotic septa and many regenerative nodules. We classified samples with a MFAP4 score ≥ 4 as having high MFAP4 expression and the remainder as having low expression. The semiquantitative scoring system was validated by automated digital image quantitation using VIS Image Analysis Software, version 2018.4 (Hoersholm, Denmark). Briefly, the entire individual liver biopsy was outlined as a region of interest, only excluding larger blood vessels and bile ducts. Next, the expression level of MFAP4 as a percentage of the total biopsy area was quantified by developing a pixel‐based algorithm to detect the immune‐positive staining signal. The algorithm was designed as a threshold‐based classification using the haematoxylin/DAB and DAB (HDAB‐DAB) feature in the image analysis software.

### 2.3 Non‐invasive liver evaluation

An experienced nurse operator (>500 scans) performed liver stiffness measurements by TE using a FibroScan 502 Touch (Echosens) according to standard procedures. We measured the commercially available ELF test (Siemens Healthcare Diagnostics, Inc) on an Advia Centaur XP according to the manufacturer's instructions (Siemens Healthcare Diagnostics, Inc). Covariation estimated from the assay control using three different concentrations ranged from 2.5% to 2.9% for tissue inhibitor of metalloproteinase‐1, 4.1% to 6.1% for hyaluronic acid and 4.0% to 5.4% for N‐terminal pro‐peptide.

### 2.4 Quantification of serum MFAP4 levels

Detection of serum MFAP4 levels was performed using an AlphaLISA technique as previously described.^16^ The experiments were performed in duplicate and sample covariance < 10% was acceptable.

### 2.5 Statistical analysis of MFAP4 in serum and liver biopsies

Summary statistics were used to describe patient characteristics. Kruskal‐Wallis test and a subsequent Dunn's test were used to test for differences in serum MFAP4 levels between fibrotic stages. We investigated which factors influenced serum MFAP4 by performing a robust multivariate linear regression analysis using stepwise elimination of insignificant factors. Serum MFAP4 levels were non‐normally distributed, and data were transformed using the natural logarithm prior to analysis. An ordinal logistic regression analysis was performed to identify variables that independently predict MFAP4 expression stage in liver biopsies. Variables with a *P* < .05 in univariate analysis were included in the final multivariate analysis. The discriminative accuracy of serum MFAP4 was tested using area under the receiver operating characteristics curve (AUROC). The cut‐offs for advanced fibrosis and cirrhosis were used if available in the literature. The remaining cut‐offs were determined by the Youden method, which maximizes the sum of sensitivity and specificity. The positive predictive value (PPV) and negative predictive value (NPV) were calculated. The Delong test was used to compare the AUROC between serum MFAP4, ELF and TE results. Diagnostic testing included both per‐protocol and intention‐to‐diagnose analyses. A risk prediction score according to serum MFAP4 levels was developed. The calibration was evaluated using Hosmer‐Lemeshow goodness‐of‐fit test with 10 quantiles together with plotting of the observed and predicted values. Results were tested in the validation cohort. The effect of MFAP4 on time‐to‐decompensation in patients with cirrhosis was illustrated by Kaplan‐Meier curves and groups were compared by log‐rank test. Cox regression analyses were performed to identify predictors of decompensation and assumption for proportional hazard was tested.

## 3 RESULTS

### 3.1 Patient characteristics

We enrolled 266 patients between April 2013 and January 2017, as outlined in the study flowchart (Supporting Information). Two patients required transfusion and intervention by reason of post‐biopsy bleeding events. Serum MFAP4 levels and ELF test were successfully measured in all patients, whereas TE measurements failed for six patients, were unreliable in another six patients or were unavailable for six cases because of equipment maintenance. The study cohort was split into a training and a validation cohort depending on whether or not patients were enrolled before (training cohort) or after (validation cohort) 31 July 2014. The characteristics of the total, training and validation cohorts are presented in Table 1. The cohorts were well‐matched according to gender and BMI, whereas the proportion of patients with advanced fibrosis was slightly less in the validation set compared to the training set.

**TABLE 1:** Characteristics of participants

Participants	All N = 266	Training set N = 153 (58%)	Validation set N = 113 (42%)	*P*‐value
Gender (male)	196 (74%)	113 (74%)	83 (74%)	.941
Age (years)	54.6 ± 10.9	56.2 ± 10.1	52.5 ± 11.7	.006
BMI (kg/m^2^)	26.7 (±5.0)	26.6 ± 5.1	26.8 ± 4.9	.834
Smoking (current)	157 (59%)	93 (61%)	64 (57%)	.610
Alcohol history				
Heavy drinking ≥ 10 years	159 (64%)	105 (69%)	54 (48%)	.040
Abstinent at inclusion	138 (42%)	74 (48.3%)	64 (57%)	.158
Daily alcohol intake in active drinkers (beverage/day)	5.9 ± 11.3	4.7 ± 5.6	7.9 ± 16.8	.121
Histological features				
Biopsy length (mm)	30.5 (±9.6)	30.5 (±9.6)	30.5 (±9.6)	
Fibrosis stage (F0/F1/F2/F3/F4)	32/93/79/17/45	15//50/46/14/28	17/43/33/3/17	
Lobular inflammation grade (0/1/2/3)	71/115/60/20	46/50/39/18	25/65/21/2	
Ballooning grade (0/1/2)	137/80/49	72/46/35	65/34/14	
Steatosis grade (0/1/2/3)	137/57/51/18	84/30/29/10	53/27/22/8	
Steatohepatitis	76 (28.6%)	51 (50%)	25 (22.1%)	.045
NAFLD activity score	2.59(±2.08)	2.72 (±2.33)	2.43(±1.71)	.156
TE	15.4 (±18.9)	17.6 (±20.7)	12.6 (±16.0)	.037
Paraclinical status				
MFAP4 (U/L)	59.5 (±47.0)	69.0 (±52.5)	46.6 (±33.4)	.000
ALT (U/L)	40.2 (±37.0)	39.1 (±31.3)	41.8 (±43.9)	.559
AST (U/L)	47.7 (±41.1)	49.1 (±39.4)	45.6 (±43.6)	.249
GGT (U/L)	204.1 (±359.7)	236.8 (±401.9)	158.6 (±286.6)	.082
AP (U/L)	106.8 (±57.4)	115.1 (±63.0)	94.6 (±46.0)	.004
INR (U/L)	1.02 (±0.14)	1.03 (±0.14)	1.00 (±0.12)	.108
Albumin (g/L)	40.5 (±4.9)	40.0 (±5.42)	41.2 (±4.0)	.022

Note

Counts are presented as N (%), continuous data are presented as mean ± SD.

Abbreviations: BMI, body mass index; TE, transient elastography; MFAP4, human microfibrillar‐associated protein 4; ALT, alanine aminotransferase; AST, aspartate transaminase; GGT, gamma‐glutamyl transferase; AP, alkaline phosphatase; INR, international normalized ratio.

### 3.2 MFAP4 serum levels in the cohorts

**FIGURE 1:**
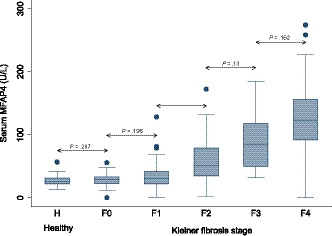
MFAP4 concentration in serum according to fibrosis stage. Boxplot of serum MFAP4 in the healthy population group and in the cohort of patients with current or prior alcohol overuse distributed according to the Kleiner fibrosis stage. The box represents the interquartile range. The whiskers indicate the highest and lowest values, and the dots represent outliers. The line across the box indicates the median value

The mean serum level for MFAP4 in the total patient cohort with prior or current alcohol overuse was 59.5 (± 47.0) U/L compared to 27.7 (± 8.8) U/L in the healthy control group that had no history of liver disease or alcohol overuse. The boxplot for serum MFAP4 in the total cohort showed that the serum level increased in relation to the Kleiner fibrosis stage as seen in Figure 1. A Kruskal‐Wallis test confirmed significant differences in serum MFAP4 levels between fibrosis stages in the total cohort (*P* = .0001). A post‐hoc analysis using Dunn´s test showed no significant difference in serum MFAP4 levels between healthy controls and patients with Kleiner fibrosis stage 0. Serum MFAP4 levels increased significantly as the fibrosis stage rose from F1 to F3 but not from F3 to F4. A multivariate analysis identified Kleiner fibrosis stage as the strongest independent predictor of serum MFAP4 levels, followed by ballooning, age, aspartate transaminase (AST) and alkaline phosphatase (AP). Serum MFAP4 was moderately correlated with the NAS score (Spearman's ρ = 0.59, *P* < .000) and the collagen proportionate area (Pearson's ρ = 0.65, *P* < .000) (Supporting Information).

### 3.3 MFAP4 expression score in liver tissue

**FIGURE 2:**
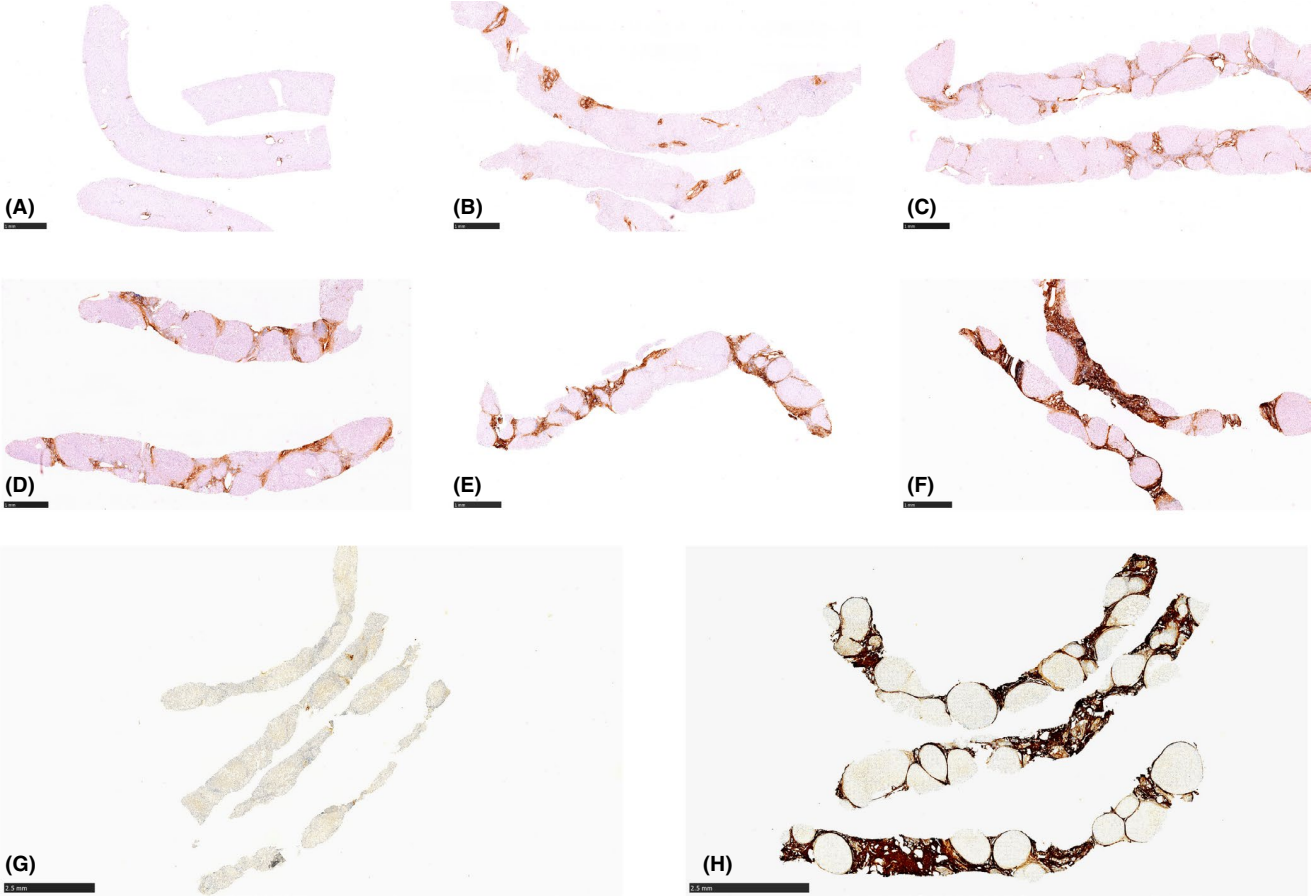
Immunohistochemical staining of MFAP4 in hepatic tissue. Representative immunohistochemical images of MFAP4 expression in the hepatic tissue in core needle biopsies. Expression of MFAP4 was semiquantitatively scored from 0 to 6. (A‐F) depicts an image of each MFAP4 expression score ranging from 1 to 6. (G) is a case of cirrhosis (Kleiner fibrosis stage 4) with a low MFAP4 expression score of 1. (H) is a case of cirrhosis (Kleiner fibrosis stage 4) with a high MFAP4 expression score of 6

To corroborate our finding of the close linkage between serum MFAP4 levels and Kleiner fibrosis stage, we scored MFAP4 expression in 116 liver biopsies having varying degrees of fibrosis to validate the presence of MFAP4 in fibrotic tissue (Supporting Information). Examples of MFAP4 scores 1‐6 are shown in Figure 2A‐F. MFAP4 was present in hepatic fibrotic tissue. In general, MFAP4 expression was more pronounced in the portal tracts relative to that in the perivenular area/zone 3. This pattern was already observed during the early stages of ALD. The presence of MFAP4 in liver tissue increased with rising Kleiner fibrosis stage in most cases (Supporting Information). MFAP4 expression score was strongly correlated to Kleiner fibrosis stage (Spearman's ρ = 0.71, *P* < .000). Figure 2 G and H show two examples of liver biopsies with histological cirrhosis (Kleiner fibrosis stage 4) that have MFAP4 expression score of 1 and 6 respectively. MFAP4 expression score was very strongly correlated to the digital image quantification of hepatic MFAP4 (Spearman's ρ = 0.91, *P* < .000). Serum MFAP4 was strongly correlated to the MFAP4 expression score (Spearman's ρ = 0.66, *P* < .000) and the digital image quantification (Pearson's ρ = 0.58, *P* < .000) (Supporting Information). We identified ballooning and age as independent predictors of MFAP4 expression score in an ordinal logistic regression model (Supporting Information). Moreover, both ballooning and MFAP4 score independently predicted serum MFAP4 levels in multivariate analyses (Data not shown).

### 3.4 Accuracy of MFAP4 for detection of advanced liver fibrosis and cirrhosis

In per‐protocol analysis, serum MFAP4 had excellent diagnostic accuracy with an AUROC of 0.88 (95% CI 0.81‐0.94) for advanced fibrosis (≥F3) and AUROC of 0.91 (95% CI 0.83‐0.98) for cirrhosis (F4) in the training cohort; similar results were seen for the validation cohort (Table 2). ROC curves for serum MFAP4 to diagnose advanced fibrosis and cirrhosis in all cohorts were created (Supporting Information). Serum MFAP4 and ELF test had similar diagnostic accuracy in terms of AUROC for both the training and validation cohorts. TE performed significantly better than serum MFAP4 in the training and total cohorts when used to detect advanced fibrosis. However, the AUROC did not significantly differ between serum MFAP4 and TE in the validation cohort, although the AUROC for TE tended to be higher than that for serum MFAP4.

**TABLE 2:** Diagnostic test results, per‐protocol and intention‐to‐diagnose analyses

Training cohort	Advanced fibrosis ≥ F3			Cirrhosis = F4		
	MFAP4	TE	ELF	MFAP4	TE	ELF
Prevalence, n (%)	42/153 (27)	37/138 (27)	42/153 (27)	28/153 (18)	23/138 (17)	28/153 (18)
AUROC (95% CI)	0.88 (0.81‐0.94)	0.95 (0.91‐0.98)	0.91 (0.85‐0.96)	0.91 (0.83‐0.98)	0.95 (0.92‐0.99)	0.95(0.91‐0.98)
AUROC vs AUROC‐MFAP4	—	*P* = .027	*P* = .348	—	*P* = .234	*P* = .257
Brier test	0.109	0.095	0.098	0.082	0.071	0.072
Hosmer‐Lemeshow test	4.27 (*P* = .832)	26.01 (*P* = .001)	6.85 (*P* = .553)	6.72 (*P* = .567)	8.22 (*P* = .412)	2.98 (*P* = .935)
Optimal cut‐off	88.7 Y	15.5 L	10.5 L	88.7 Y	19.7 L	11.1 Y
Correctly classifies n (%)	132 (86)	124 (90)	132 (86)	132 (86)	123 (89)	139 (91)
TP/FP/FN/TN	32/11/10/100	32/9/5/92	34/13/8/98	25/18/3/107	22/14/1/101	25/11/3/114
Sensitivity (%)	76 (61‐88)	87 (71‐96)	81 (66‐91)	89 (72‐98)	96 (78‐100)	89 (72‐98)
Specificity (%)	90 (83‐95)	91 (84‐96)	88 (81‐94)	86 (78‐91)	88 (80‐93)	91 (85‐96)
PPV (%)	74 (59‐87)	78 (62‐89)	72 (57‐84.4)	58 (42‐73)	61 (44‐77)	69 (52‐84)
NPV (%)	91 (84‐96)	95 (88‐98)	93 (86‐97)	97 (92‐99)	99 (95‐100)	97 (93‐100)
Pretest odds	0.37	0.37	0.37	0.22	0.20	0.22
LR (+)	7.69 (4.28‐13.8)	9.71 (5.14‐18.3)	6.91 (4.06‐11.8)	6.2 (3.97‐9.69)	7.86 (4.77‐12.9)	10.1 (5.69‐18.1)
LR (−)	0.26 (0.15‐0.46)	0.15 (0.07‐0.34)	0.22 (0.12‐0.40)	0.13 (0.04‐0.37)	0.05 (0.01‐0.24)	0.12 (0.04‐0.34)
Validation cohort	MFAP4	TE	ELF	MFAP4	TE	ELF
Prevalence n (%)	20/113 (18)	18/110 (16)	20/113 (18)	17/113 (15)	15/110 (14)	17/113 (15)
AUROC (95% CI)	0.92 (0.83‐1.00)	0.98 (0.96‐1.00)	0.94 (0.88‐0.99)	0.91 (0.79‐1.00)	0.97 (0.95‐1.00)	0.92 (0.85‐0.98)
AUROC vs AUROC‐MFAP4	—	*P* = .247	*P* =.84	—	*P* = .255	*P* = .865
Brier test	0.061	0.047	0.069	0.062	0.058	0.073
Hosmer‐Lemeshow test	14.86 (*P* = .062)	16.01 (*P* = .042)	9.17 (*P* = .328)	14.76 (*P* = .064)	15.13 (*P* = .057)	5.30 (*P* = .725)
Cut‐off	88.7 Y	15.5 L	10.5 L	88.7 Y	19.7 L	11.1 Y
Correctly classifies n (%)	104 (92)	108 (98)	100 (88)	105 (93)	102 (93)	101 (89)
TP/FP/FN/TN	11/0/9/93	18/2/0/90	14/7/6/86	10/1/7/95	12/5/3/90	10/5/7/91
Sensitivity (%)	55 (32‐77)	100 (82‐100)	70 (46‐88)	59 (33‐82)	80 (52‐96)	59 (33‐82)
Specificity (%)	100 (96‐100)	98 (92‐100)	93 (85‐97)	99 (94‐100)	95 (88‐98)	95 (88‐98)
PPV (%)	100 (72‐100)	90 (68‐99)	67 (43‐85)	91 (59‐100)	71 (44‐90)	67 (38‐88)
NPV (%)	91 (84‐96)	100 (96‐100)	94 (86‐98)	93 (86‐97)	97 (91‐99)	93 (86‐97)
Pretest odds	0.22	0.20	0.22	0.18	0.16	0.18
LR (+)	(High)	46 (11.7‐181)	9.3 (4.31‐20)	56.5 (7.72‐413)	15.2 (6.24‐37)	11.3 (4.41‐29)
LR (−)	0.45 (0.28‐0.73)	0	0.32 (0.17‐0.63)	0.42 (0.24‐0.74)	0.21 (0.08‐0.58)	0.43 (0.25‐0.77)
Total cohort	MFAP4	TE	ELF	MFAP4	TE	ELF
Prevalence n (%)	62/266 (23)	55/248 (22)	62/266 (23)	45/266 (17)	38/248 (15)	45/266 (17)
AUROC (95% CI)	0.90 (0.85‐0.95)	0.96 (0.94‐0.99)	0.92 (0.88‐0.96)	0.90 (0.84‐0.96)	0.96 (0.94‐0.98)	0.93 (0.90‐0.97)
AUROC vs AUROC‐MFAP4	—	*P* = .011	*P* = .440	—	*P* = .061	*P* = .314
Brier test	0.0922	0.076	0.086	0.079	0.102	0.072
Hosmer‐Lemeshow test	7.28 (*P* = .506)	50.13 (*P* = .000)	10.85 (*P* = .210)	14.09 (*P* = .080)	23.18 (*P* = .003)	3.32 (*P* = .912)
Cut‐off	62.0 Y	15.5 L	10.5 L	60.3 Y	19.7 L	10.1 Y
Correctly classifies n (%)	224 (84)	232 (94)	232 (87)	214 (80)	225 (91)	218 (82)
TP/FP/FN/TN	53/33/9/171	50/11/5/182	48/20/14/184	42/49/3/172	34/19/4/191	42/45/3/176
Sensitivity (%)	86 (74‐93)	91 (80‐97)	77 (64‐87)	93 (82‐99)	90 (75‐97)	93 (82‐99)
Specificity (%)	84 (78‐89)	84 (90‐97)	90 (85‐94)	77.8 (72‐99)	91 (86‐95)	80 (74‐85)
PPV (%)	62 (51‐72)	82 (70‐91)	71 (58‐81)	46 (36‐57)	64 (50‐77)	48 (37‐59)
NPV (%)	95 (91‐98)	97 (94‐99)	93 (88‐96)	98 (95‐100)	98 (95‐99)	98 (95‐100
Pretest odds	0.30	0.28	0.30	0.20	0.18	0.20
LR (+)	5.28 (3.8‐7.34)	16 (8.93‐28.5)	7.9 (5.1‐12.2)	4.21 (3.25‐5.45)	9.89 (6.35‐15.4)	4.58 (3.49‐6.02)
LR (−)	0.17 (0.09‐0.32)	0.10 (0.04‐0.22)	0.25 (0.16‐0.40)	0.09 (0.03‐0.26)	0.12 (0.05‐0.29)	0.08 (0.03‐0.25)
Intention‐to‐diagnose analysis	MFAP4	TE	ELF	MFAP4	TE	ELF
Prevalence n (%)	20/113 (18)	20/113 (18)	20/113 (18)	17/113 (15)	17/113 (15)	17/113 (15)
AUROC (95% CI)	0.92 (0.83‐1.00)	0.93 (0.83‐1.00)	0.94 (0.88‐0.99)	0.91 (0.79‐1.00)	0.91 (0.80‐1.00)	0.92 (0.85‐0.98)
AUROC vs AUROC‐MFAP4	—	*P* = .918	*P* = .84	—	*P* = .924	*P* = .865
Brier test	0.061	0.063	0.069	0.062	0.067	0.073
Hosmer‐Lemeshow test	14.86 (*P* = .062)	13.05 (*P* = .110)	9.17 (*P* = .328)	14.76 (*P* = .064)	12.43 (*P* = .133)	5.30 (*P* = .725)
Cut‐off	88.7 Y	15.5 L	10.5 L	88.7 Y	19.7 L	11.1 Y
Correctly classifies n (%)	104 (92)	109 (96)	100 (88)	105 (93)	103 (91)	101 (89)
TP/FP/FN/TN	11/0/9/93	19/3/1/90	14/7/6/86	10/1/7/95	13/6/4/90	10/5/7/91
Sensitivity (%)	55 (32‐77)	95 (75‐100)	70 (46‐88)	59 (33‐82)	77 (50‐93)	59 (33‐82)
Specificity (%)	100 (96‐100)	97 (91‐99)	93 (85‐97)	99 (94‐100)	94 (87‐98)	95 (88‐98)
PPV (%)	100 (72‐100)	86 (65‐97)	67 (43‐85)	91 (59‐100)	68 (43‐87)	67 (38‐88)
NPV (%)	91 (84‐96)	99 (94‐100)	94 (86‐98)	93 (86‐97)	96 (90‐99)	93 (86‐97)
Pretest odds	0.22	0.22	0.22	0.18	0.19	0.18
LR (+)	(High)	29.4 (9.63‐90.1)	9.3 (4.31‐20)	56.5 (7.72‐413)	12.2 (5.4‐28)	11.3 (4.41‐29)
LR (−)	0.45 (0.28‐0.73)	0.05 (0.01‐0.35)	0.32 (0.17‐0.63)	0.42 (0.24‐0.74)	0.25 (0.11‐0.59)	0.43 (0.25‐0.77)

Note

Y in the cut‐off indicates that the value was identified by optimizing the Youden index and an L indicates that the value was identified based on published literature.

Abbreviations: AUROC, area under the receiver operating characteristics curve; TP, true positive; FP, false positive; FN, false negative; TN, true negative; PPV, positive predictive value; NPV, negative predictive value; LR, likelihood ratio.

The optimal cut‐offs for detecting advanced fibrosis and cirrhosis were both 88.7 U/L for serum MFAP4 in the training cohort when cut‐offs were optimized by the Youden index. The cut‐off for TE was set to 15.5 kPa and 19.7 kPa for advanced fibrosis and cirrhosis, respectively, based on our previously published results.^15^, ^26^ The cut‐off for ELF to detect advanced fibrosis was set to 10.5 based on previously published values and a cut‐off of 11.1 to detect cirrhosis was established by optimizing the Youden index in the training cohort.^15^ Serum MFAP4 had a high NPV for advanced fibrosis and cirrhosis (91% and 97% respectively) and a moderate PPV (74% for advanced fibrosis and 58% for cirrhosis) in the training cohort. In the validation cohort, serum MFAP4 also had high NPV for the exclusion of advanced fibrosis (91%) and cirrhosis (93%). The PPV in the validation cohort was 100% for advanced fibrosis and 91% for cirrhosis, which aligns with the training cohort results. Out of the total cohort, 11 and 19 patients were false positive for advanced fibrosis and cirrhosis, respectively, when a cut‐off of 88.7 U/L was applied (Table 2). In a logistic regression model that considered AST, AP, ballooning, age and biopsy length, the only risk factors for misclassification because of false positive were ballooning (OR 2.66, 95% CI: 1.18‐6.01, *P* = .018) and AST (1.02, 95% CI: 1.01‐1.02, *P* = .001).

**FIGURE 3:**
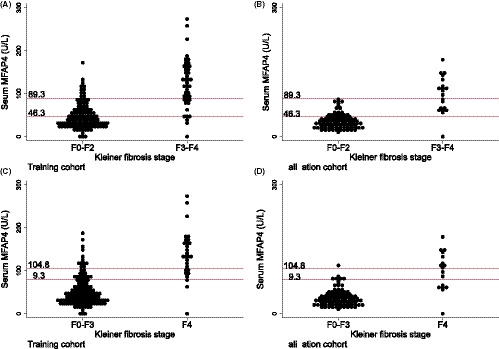
Distributional plots of serum MFAP4 in the training and validation cohorts. Distributional plots of serum MFAP4 according to advanced fibrosis and cirrhosis in the training and validation cohorts. Rule‐in and rule‐out cut‐offs are marked with a red line. A&B: MFAP4 > 89.3 (U/L) can be used to rule in advanced fibrosis and a MFAP4 < 46.3 (U/L) can be used to rule out advanced fibrosis in the training (A) and the validation cohort (B). C&D: MFAP4 > 104.8 (U/L) can be used to rule in cirrhosis and a MFAP4 < 79.3 (U/L) can be used to rule out cirrhosis in the training (C) and the validation cohort (D)

To optimize stratification of patients according to MFAP4 serum level, we determined rule‐in and rule‐out cut‐offs for advanced fibrosis and cirrhosis by setting specificity (to rule‐in) or sensitivity (to rule‐out) to 90%. Serum MFAP4 levels above the threshold suggested a high likelihood of ALD diagnosis, and vice versa. Values that fell between the cut‐offs would warrant further investigation (Figure 3 and Supporting Information).

**FIGURE 4:**
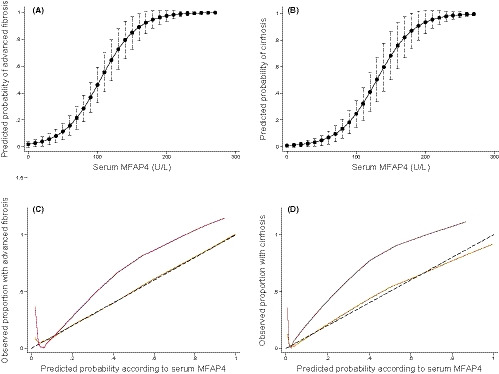
Risk prediction and calibration curves according to serum MFAP4. (A and B) Risk prediction curves to evaluate the probability of advanced fibrosis and cirrhosis constructed by logistic regression with serum concentration of MFAP4 in the test cohort. (C and D) Calibration slopes for MFAP4 in the test (yellow) and validation cohort (red). The slopes graph the agreement between predicted probability of advanced fibrosis/cirrhosis on the *x*‐axis and observed proportion with advanced fibrosis/cirrhosis on the y‐axis. The black dashed line represents perfect calibration, with 100% agreement

Both risk prediction plots and calibration plots are shown in Figure 4. The calibration of serum MFAP4 was good for both advanced fibrosis (Hosmer‐Lemeshow 4.27, *P* = .83) and cirrhosis (Hosmer‐Lemeshow 6.72, *P* = .57). Although the serum MFAP4 risk model developed in the test cohort tended to underestimate the observed risk of advanced fibrosis and cirrhosis in the validation cohort, the overall difference between the predicted and the observed risk in the validation cohort did not differ significantly for either advanced fibrosis (Hosmer‐Lemeshow 14.86, *P* = .06) or cirrhosis (Hosmer‐Lemeshow 14.76, *P* = .06).

### 3.5 Intention‐to‐diagnose analysis

To consider the impact of non‐evaluable results, we performed an intention‐to‐diagnose analysis using the cut‐off values from the training cohort to compare the diagnostic potential of serum MFAP4, ELF and TE. Unreliable results, which did not meet quality criteria of the diagnostic test, were included in the analysis, whereas those caused by equipment maintenance were excluded. When a test failed and no measurement was available, the result was coded as false negative or false positive depending on the Kleiner fibrosis stage. The analysis negatively impacted the diagnostic accuracy of TE, whereas that for serum MFAP4 and ELF remained unchanged (Table 2). The diagnostic accuracy of serum MFAP4, whether evaluated by AUROC or Brier score, was similar to that of TE and ELF in the intention‐to‐diagnose analysis.

### 3.6 Relationship between MFAP4 and time‐to‐decompensation in cirrhosis

Cirrhotic patients with high MFAP4 expression in hepatic tissue had significantly larger spleens and tended to have lower platelet counts compared to cirrhotic patients who had low MFAP4 expression (Supporting Information). As these findings potentially reflect differences in the portal pressure between the groups, we subsequently evaluated whether MFAP4 could be a prognostic marker of portal hypertension‐related hepatic decompensation in patients with cirrhosis. Follow‐up data for all 45 patients with cirrhosis were available for analysis. The primary composite endpoint was a hepatic decompensation episode that involved occurrence of any of the following events during the follow‐up period: development of varices, ascites, hepatic encephalopathy, hepatocellular carcinoma, hepatorenal syndrome, progression of ascites or varices. Patients were followed from inclusion until 1 July 2017. The median follow‐up period was 452 days (range: 2‐1,468). During follow‐up, 24 patients developed the primary endpoint of a hepatic decompensation episode. Two patients died as a result of causes unrelated to liver disease and were censored at the time of death. The 45 cirrhosis patients had early stage cirrhosis with a median Child‐Pugh score of 6 (5‐9) and were either Child‐Pugh class A (n = 30) or B (n = 15). Among the 15 class B patients, nine, three and three scored as B7, B8 and B9 respectively. Seven patients had minimal ascites that were detectable only by ultrasound and nine patients had grade 1 varices.

**FIGURE 5:**
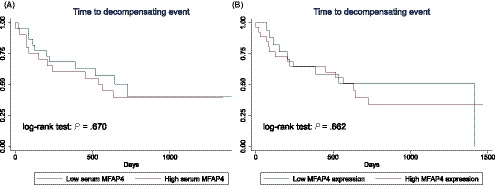
Time to hepatic decompensation in cirrhosis according to MFAP4. A, Kaplan‐Meier plot of hepatic decompensation probability in patients with cirrhosis according to high or low serum concentration of MFAP4. Disease progression did not differ between groups by log‐rank test (*P* = .670). B, Kaplan‐Meier plot of hepatic decompensation probability in patients with cirrhosis according to high or low expression of MFAP4 in hepatic tissue. Disease progression did not differ between groups by log‐rank test (*P* = .661)

Patients were divided into two groups depending on whether they had a MFAP4 serum level below or above the median value of 123.4 (U/L). The median time‐to‐decompensation when MFAP4 levels were below the median value was 727 days and 566 days when the threshold exceeded the median value. The Kaplan‐Meier plots indicated equal decompensation rates comparing patients above or below the median serum MFAP4 value as seen in Figure 5. A Cox regression model indicated that the Child‐Pugh score (HR 1.41, 95% CI: 1.04‐1.93, *P* = .03) and abstinence at inclusion (HR 0.36, 95% CI 0.16‐0.83, *P* = .02) were predictors of disease decompensation in univariate analysis, but serum MFAP4 was not (HR 1.01, 95% CI 1.00‐1.02, *P* = .128). Among the 27 patients having high MFAP4 levels in liver biopsies (score ≥ 4), the median time‐to‐decompensation was 633 days, whereas the 18 patients who had low MFAP4 expression (score ≤ 3) had a mean time of 1,411 days. The Kaplan‐Meier plots likewise indicated equal decompensation rates comparing patients with a high or low MFAP4 expression as seen in Figure 5. In a Cox regression analysis, high MFAP4 expression was not predictive of decompensation (HR 1.20, 95% CI: 0.53‐2.75, *P* = .663).

## 4 DISCUSSION

Owing to the high prevalence of alcohol overuse there is a need for biomarkers that can penetrate into the primary care setting where the majority of patients with ALD are seen. MFAP4 has the advantage that it can be added on top of the standard liver function test widely performed during standard workup in primary care and does not require implementation and maintenance of expensive equipment such as elastography. Serological fibrosis assessment using patented markers remains costly and new biomarkers as MFAP4 or others may be an attractive less expensive alternative.

The results of this biopsy‐controlled study with a validation cohort strongly support the clinical usefulness of MFAP4 as a blood‐borne marker for assessing fibrosis in ALD. We found that hepatic expression of MFAP4 is upregulated in fibrotic tissue and that MFAP4 serum levels increase with the severity of fibrosis. We showed that serum MFAP4 has excellent diagnostic accuracy similar to that of ELF and TE, which are the existing benchmarks for non‐invasive detection of fibrosis in ALD.^14^, ^15^ Finally, we demonstrated that MFAP4 was not a strong prognostic marker for hepatic decompensation in patients with early stage cirrhosis. Our results are in line with previous findings consistently reporting a favourable diagnostic accuracy of serum MFAP4 for detection of fibrosis in cohorts with HCV.^22^, ^23^ MFAP4 has not been previously assessed as a prognostic marker in liver disease. Increasing amounts of evidence support that most complications in cirrhosis develop as part of a decompensating event or as acute‐on‐chronic liver failure.^28^ These events are frequently triggered by inflammation rather than progression of fibrosis.^29^ This dynamic natural history of cirrhosis likely cannot be reflected by a single fibrosis marker and may explain why MFAP4 failed as a predictor of decompensation in our study.^28^

Ballooning was independently associated with both hepatic expression and serum level of MFAP4, suggesting a potential role for ballooning in the upregulation of MFAP4 expression in fibrotic tissue and subsequent release to the blood stream. Consistent with these findings, we identified ballooning as a risk factor for false classification of cirrhosis.

Although our study was specifically designed to evaluate the diagnostic accuracy of serum MFAP4, we do note some strengths and limitations. Our cohort included 266 patients who had varying degrees of alcohol consumption that covered the full fibrotic spectrum of ALD from no fibrosis to fully developed cirrhosis. By including patients recruited from primary care, we increased the generalizability of our results. Likewise, we chose to include patients with obesity or features of metabolic syndrome as these conditions often co‐exist with alcohol overuse and reflect daily clinical practice. Patients who had obvious ascites and large varices were excluded as these patients rarely require further diagnostic workup in the form of a liver biopsy to secure a diagnosis of cirrhosis. No patients in the cohort suffered from severe pulmonary or cardiovascular diseases and there was only one case of (chronic) pancreatitis. Such conditions may affect MFAP4 serum levels and could potentially impact the diagnostic accuracy of MFAP4 in a clinical setting.^16^, ^30^, ^31^ The study cohort was split into a training and a validation cohort by date as this leads to temporal validation.^32^ MFAP4 maintained a high diagnostic accuracy, despite differences in clinical phenotypes and disease prevalence between the cohorts. However, differences in disease prevalence leading to spectrum bias should still be considered when generalizing results.^26^ Further, the prognostic analyses should be interpreted with some caution as the follow‐up time was relatively short for patients with early stage cirrhosis. However, since more than half of the patients met the primary endpoint during the follow‐up period, the high event rate compensated for the short follow‐up period and justified the time‐to‐decompensation analysis.

In conclusion, serum MFAP4 is a novel, highly accurate marker for assessing ALD‐induced fibrosis with similar diagnostic accuracy as TE and ELF test.

## 5 TRIAL REGISTRATION NUMBER

The studies are registered in the Odense Patient Data Exploratory Network (OPEN) under study identification numbers OP_040 (https://open.rsyd.dk/OpenProjects/da/openProject.jsp?openNo=40) and OP_239 (https://open.rsyd.dk/OpenProjects/openProject.jsp?openNo=239&lang=da).

## CONFLICT OF INTEREST

AS, UH and GLS are inventors of US Patent No. 9,988,442 and EP17199552.5 owned by University of Southern Denmark. Remaining authors have nothing to declare.

## Supporting information

Supplementary Material
