# Supplementary material for: Prediction of liver fibrosis severity in alcoholic liver disease by human microfibrillar‐associated protein 4
Source: Liver Int. 2020 May 10;40(7):1701–12. doi: 10.1111/liv.14491 (PMC7383987; doi:10.1111/liv.14491)
Supplement: Supplementary file 1 — Supplementary Material [file LIV-40-1701-s001.docx]

**Supporting Information**

1. Figure: Study flowchart
2. Table: Association of study variables with serum levels of MFAP4 in a robust linear regression model
3. Table: Relation between Kleiner fibrosis stage and hepatic MFAP4 expression
4. Figure: Serum MFAP4 according to the hepatic expression
5. Figure: Serum MFAP4 according to percentage of biopsy area
6. Table: Association of study variables with hepatic MFAP4 expression score in an ordered logistic regression model
7. MFAP4 expression and surrogate markers of portal pressure
8. Figure: Receiver Operating Characteristic for MFAP4 to diagnose advanced fibrosis and cirrhosis
9. Table: Diagnostic values for rule-in and rule-out cutoffs
10. Table: Liver-Fibro STARD checklist
11. Figure: Relation between serum MFAP4 and nonalcoholic fatty liver disease activity (NAS) score
12. Figure: Serum MFAP4 according to collagen proportionate area

1. Figure: Study flowchart

Fulfilled inclusion and exclusion criteria

(N=520)

Excluded (n= 246)

-Declined to participate (n=219)

-Liver biopsy not indicated due to liver stiffness <6 (n=19)

-Hospitalized prior to inclusion (n=4)

-Died prior to inclusion (n=2)

-Diagnosed with cancer prior to inclusion (n=2)

274 patients with liver biopsy in the cohort

Excluded after liver biopsy (n=8)

-Severe alcoholic hepatitis (n=1)

-No blood samples available (n=4)

-Biopsy length <10 mm n=3)

266 patients with liver biopsy and blood samples in the final cohort

2. Table: Association of study variables with serum level of MFAP4 in a robust linear regression model

| Variable | Univariate analysis  Coefficient (95% CI) | *P* | Multivariate analysis  Coefficient (95% CI) | *P* |
| --- | --- | --- | --- | --- |
|  |  |  |  |  |
| Gender* | 1.063 (0.867-1.304) | .555 |  |  |
| Age | 1.024(1.017-1.032) | .000 | 1.014 (1.010-1.020) | .000 |
| BMI | 1.004(0.986-1.022) | .663 |  |  |
| Smoking | Smoker: 0.774 (0.593-1.011)  Ex-smoker:1.122 (0.836-1.506) | .061  .442 |  |  |
| Abstinent at inclusion | 0.850 (0.712-1.015) | .073 |  |  |
| Daily alcohol intake (beverage/day) | 1.005 (0.994-1.016 | .342 |  |  |
| Creatinin | 0.989 (0.984-0.995) | .001 |  |  |
| ALT | 1.000((0.998-1.003) | .804 |  |  |
| AP | 1.006((1.005-1.007) | .000 | 1.001 (1.000-1.003) | .005 |
| AST | 1.007(1.005-1.009) | .000 | 1.003 (1.002-1.004) | .000 |
| Bilirubine | 1.036(1.028-1.044) | .000 |  |  |
| GGT | 1.001 (1.000-1.001) | .000 |  |  |
| Fibrosis stage (F0/F1/F2/F3/F4) | F1 1.074 (0.883-1.306)  F2 1.815 (1.486-2.217)  F3 2.861 (2.298-3.811)  F4 4.286 (3.437-5.346) | .473  .000  .000  .000 | F1 0.982 (0.786-0.149)  F2 1.375 (1.136-1.665)  F3 2.065 (1.578-2.703)  F 4 2.577 (2.031-3.271) | .883  .001  .000  .000 |
| Lobular inflammation grade (0/1/2/3) | I1 1.645 (1.365-1.984)  I2 2.173 (1.749-2.700)  I3 3.742 (2.735-5.119) | .000  .000  .000 |  |  |
| Ballooning grade (0/1/2) | B1 1.655 (1.422-1.927)  B2 3.344 (2.794-4.002) | .000  .000 | B1 1.129 (0.987-1.291)  B2 1.273 (1.050-1.544) | .077  .014 |
| Steatosis grade (0/1/2/3) | S1 1.472 (1.174-1.846)  S2 1.261 (0.998-1.593)  S3 1.434 (1.008-2.038) | .001  .052  .045 |  |  |

*Male gender used as reference

Abbreviations: BMI : body mass index , ALT: alanine transaminase, AP: alkaline phosphatase, AST: apartate transaminase GGT: gamma-glutamyltransferase

3. Table: Relation between Kleiner fibrosis stage and hepatic MFAP4 expression

|  | Digital image quantification* | Expression score** | | | | | |
| --- | --- | --- | --- | --- | --- | --- | --- |
| Kleiner fibrosis stage | % | 1 | 2 | 3 | 4 | 5 | 6 |
| 0 | 0.6(0.1-1.4) | 10 | 0 | 0 | 0 | 0 | 0 |
| 1 | 0.6(0.2-3.1) | 20 | 2 | 0 | 0 | 0 | 0 |
| 2 | 1.7(0.4-7.7) | 6 | 14 | 6 | 0 | 0 | 0 |
| 3 | 2.8(0.2-32.1) | 4 | 3 | 2 | 4 | 0 | 0 |
| 4 | 10.1(0.2-44.4) | 5 | 8 | 5 | 10 | 12 | 5 |

*Percentage of liver biopsy area covered by MFAP4

** Semiquantitative assessment of the hepatic expression of MFAP4

4. Figure: MFAP4 serum level according to its expression in the hepatic tissue

Scatterplot of serum level and hepatic expression score of MFAP4 in 116 patients with prior or current alcohol overuse. The MFAP4 expression in the hepatic tissue was semiquantitatively scored from 0 to 6.

5. Figure: Serum MFAP4 according to percentage of biopsy area


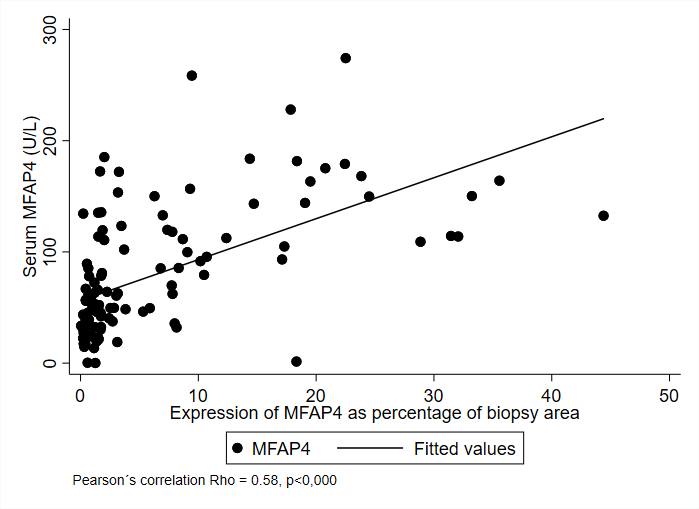


Scatterplot of serum levels and hepatic expression of MFAP4. Automated digital image quantification was used to estimate the percentage of the total biopsy area covered by MFAP4 expression.

6. Table: Association of study variables with hepatic MFAP4 expression score in an ordered logistic regression model

| Variable | Univariate analysis  Odds Ratio (95% CI) | *P* | Multivariate analysis  Odds Ratio (95% CI) | *P* |
| --- | --- | --- | --- | --- |
|  |  |  |  |  |
| Gender | 1.06 (0.50-2.24) | .872 |  |  |
| Age | 1.07 (1.03-1.11) | .000 | 1.04 (1.00-1.08) | .042 |
| BMI | 1.00 (0.94-1.07) | .943 |  |  |
| Smoking | Smoker: 0.92 (0.31-2.70)  Ex-smoker: 0.92 (0.30-2.83) | .877  .880 |  |  |
| Abstinent at inclusion | 0.99 (0.51-1.93) | .981 |  |  |
| Daily alcohol intake (beverage/day) | 1.02 (0.95-1.10) | .524 |  |  |
| Lobular inflammation grade (0/1/2/3) | I1 4.00 (1.52-10.57)  I2 4.54 (1.54-13.35)  I3 5.09 (1.38-18.78) | .005  .006  .014 | I1 1.38 (0.48-3.92)  I2 0.83 (0.23-2.93)  I3 0.48 (0.10-2.21) | .550  .770  .346 |
| Ballooning grade (0/1/2) | B1 8.85 (3.53-22.16)  B2 15.76 (6.27-39.67) | .000  .000 | B1 7.81 (2.85-21.4)  B2 17.23 (5.64-52.64) | .000  .000 |
| Steatosis grade (0/1/2/3) | S1 1.25 (0.60-2.61)  S2 0.95 (0.36-2.48)  S3 0.71 (0.06-9.06) | .552  .909  .792 |  |  |

*Male gender used as reference

Abbreviations: CI: confidence interval, BMI: body mass index

7. MFAP4 expression and surrogate markers of portal pressure

A: B:

A: Box plot of spleen diameter in patients with cirrhosis according to high or low expression of MFAP4 in the hepatic tissue. Difference in diameter is statistical significant by Mann-Whitney test *P* =.018.

B: Box plot of platelet count in patients with cirrhosis according to high or low expression of MFAP4 in the hepatic tissue. Difference in platelet count is borderline statistical significant by Mann-Whitney test *P* =.052

8. Figure: Receiver Operating Characteristic for MFAP4 to detect advanced fibrosis and cirrhosis


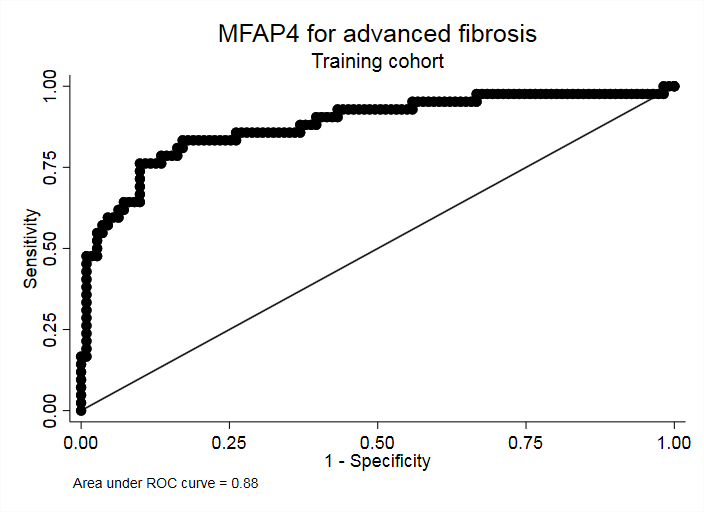

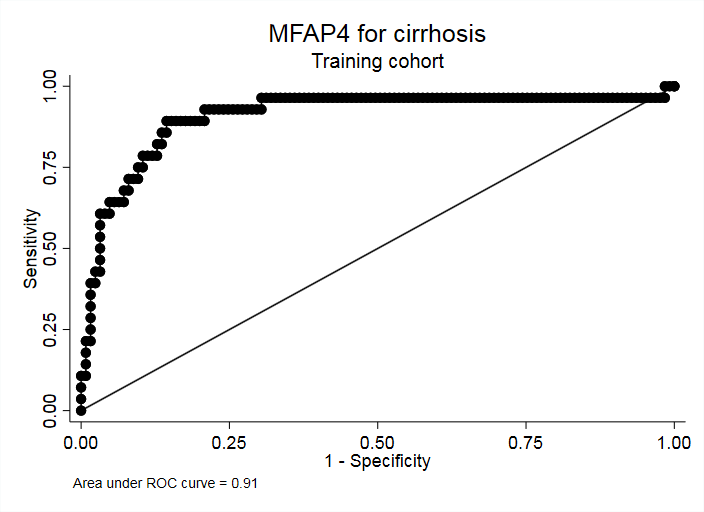


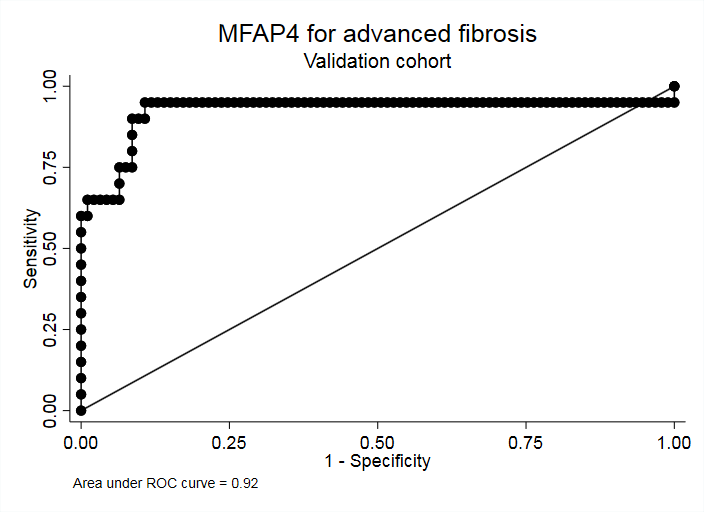

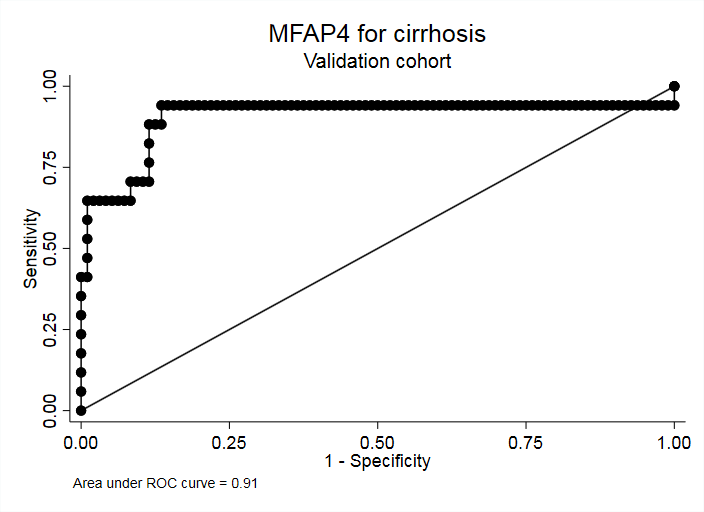


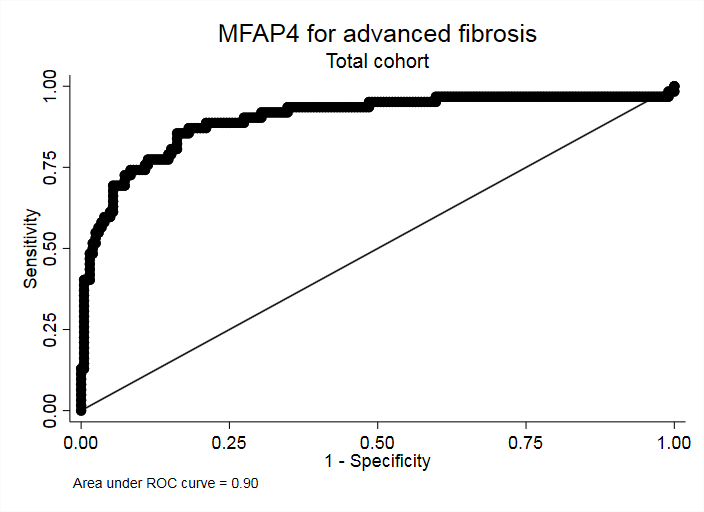

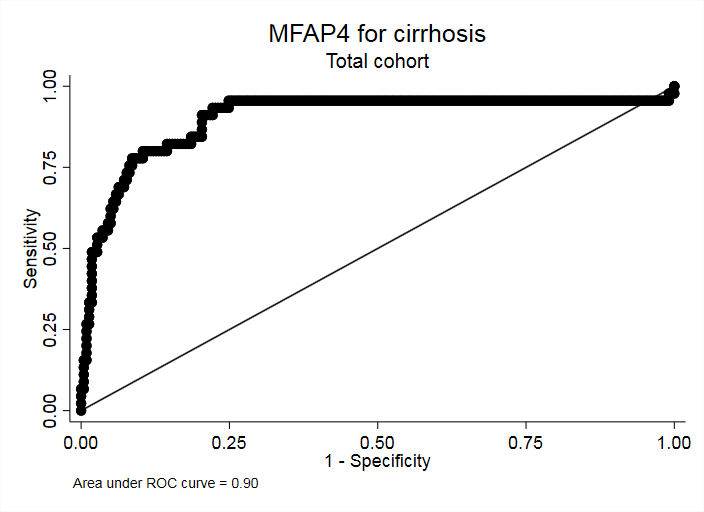


9. Table: Diagnostic values for rule-in and rule-out cutoffs

| Decision table to rule-in or rule-out advanced fibrosis (≥F3) and cirrhosis (F4) by means of low and high cutoff values for serum MFAP4 | | | | | | |
| --- | --- | --- | --- | --- | --- | --- |
| Training cohort | | | | | | |
| Diagnosis | Decision | Cutpoint (U/L) | Sens (%) | Spec (%) | PPV (%) | NPV (%) |
| Advanced fibrosis (≥F3) | Rule out | 46.3 | 91(77-97) | 60(51-70) | 46(35-58) | 94(86-98) |
|  | Rule in | 89.3 | 76(61-88) | 90(83-95) | 74(59-87) | 91(84-96) |
| Cirrhosis (F4) | Rule out | 79.3 | 93(77-99) | 79(71-86) | 50(36-64) | 98(93-100) |
|  | Rule in | 104.8 | 75(55-89) | 90(84-95) | 64(45-80) | 94(88-98) |
| Validation cohort | | | | | | |
| Diagnosis | Decision | Cutpoint (U/L) | Sens (%) | Spec (%) | PPV (%) | NPV (%) |
| Advanced fibrosis (≥F3 | Rule out | 46.3 | 95(75-100) | 81(71-88) | 51.4(34-68) | 99(93-100) |
|  | Rule in | 89.3 | 55(32-77) | 100(96-100) | 100(72-100) | 91(84-96) |
| Cirrhosis (F4) | Rule out | 79.3 | 65(38-86) | 94(87-98) | 65(38-86) | 94(87-98) |
|  | Rule in | 104.8 | 53(28-77) | 99(94-100) | 90(56-100) | 92(85-97) |
| Abbreviations: Sens: sensibility, Spec: specificity, PPV: positive predictive value, NPV: negative predictive value | | | | | | |

10. Table: Liver-Fibro STARD checklist

**LIVER-FIBROSTARD CHECKLIST**

The Liver-FibroSTARD checklist summarizes the important information that must be present in the manuscripts of diagnostic studies on non-invasive tools for liver fibrosis evaluation. Compared to STARD, the Liver-FibroSTARD checklist includes 2 additional items (#12 and #26) and 44 sub-items. The sub-items correspond to those proposals that clearly depicted, within the items, each of the particular features of diagnostic studies on liver fibrosis tests. Finally, Liver-FibroSTARD presents as a complementary module of the STARD checklist.

*Some items or sub/items include several criteria; major criteria are indicated by an asterisk (*). Example: item #3: “The study population: The inclusion and exclusion criteria*, setting, and locations* where data were collected”. If a major item is missing, the corresponding criterion has to be rated absent. Some items/sub-items (#12.1 and #23.1, #13.10 and #22.2) are redundant since they can be found in different locations of the article*

| **TITLE/ABSTRACT/KEYWORDS**  **1. Identify the article as a study of diagnostic accuracy (recommend MeSH heading “sensitivity and specificity”).** | |  | |
| --- | --- | --- | --- |
| *1.1. Identify the article, especially in the title, as a study of the diagnostic performance of liver fibrosis/cirrhosis biomarker(s)/test(s).* | | Title page | |
| *1.2. Recommended key words (choose the most appropriate): “liver fibrosis”, “cirrhosis”, “diagnosis”, “biomarker”, “diagnostic test”, “noninvasive diagnosis”.* | | Title page | |
| **INTRODUCTION**  **2. State the research questions or study aims, such as estimating diagnostic accuracy or comparing accuracy between tests or across participant groups.** | |  | |
| *In study aims, specify:* | | | |
| *2.1. If the aim is to identify new marker(s)/develop new test(s), or to evaluate published marker(s)/test(s).* | | p.8 | |
| *2.2. Whether the study is performed in a single or multiple cause(s) of chronic liver disease.* | | p.9 | |
| *2.3. The reference used for fibrosis diagnosis in the study.* | | p.9-10 | |
| *2.4. The diagnostic target used as the primary aim of the study and, if appropriate, other diagnostic targets used as secondary aims.* | | p.10-11 | |
| **METHODS** | | *Describe:* | |
| Participants  **3. The study population: The inclusion and exclusion criteria*, setting, and locations* where data were collected.** |  | | *p.9* |
| **4. Participant recruitment: Was recruitment based on presenting symptoms, results from previous tests, or the fact that the participants had received the index tests or the reference standard?** | | | |
| *4.1. State if healthy subjects without chronic liver disease are included or not in the study.* | | p.9  The study included 50 healthy participants in which the biomarker MFAP4 was measured | |
| *4.2. State if patients were selected by one abnormal or several discordant fibrosis test(s).* | | p.10  Selection for the study was based on risk profile | |
| *4.3. State if patients were selected according to the availability of reference or index test(s) result(s).* | | No | |
| **5. Participant sampling: Was the study population a consecutive series of participants defined by the selection criteria in item 3 and 4? If not, specify how participants were further selected.** | | Yes | |
| **6. Data collection: Was data collection planned before the index test and reference standard were performed (prospective study) or after (retrospective study)?** | | | |
| *6.1. The chronology between patient inclusion*, data collection (reference/index tests)*, and data analysis is well described.* | | p.9  Patients were prospectively enrolled | |
| *6.2. Has the study population been previously used/published for the evaluation of the studied fibrosis test(s)?* | | The diagnostic accuracy of MFAP4 has never been evaluated in the study population. However the cohort has previously been used to evaluate the diagnostic accuracy of transient elastography and the ELF test. | |
| Test methods  **7. The reference standard and its rationale** |  | | p.10-11  Liver biopsy, the gold standard |
| **8. Technical specifications of material and methods involved including how and when measurements were taken, and/or cite references for index tests and reference standard.** | | | |
| *For the reference and index test(s), specify characteristics with sufficient detail to permit exact reoperation, when appropriate:* | | | |
| *8.1. Center: standardization of procedures across centers.* | | P.9-12  Singlecenter study, investigations were performed according to standard operating procedures | |
| *8.2. Patient: fasting conditions*, time, posture, etc. (give information about the influence of conditions on the intra-individual variability).* | | p.10 | |
| *8.3. Delay: time interval between reference and index test(s).* | | p.10  All procedures and investigations were performed on the same day | |
| *8.4. Material: technical specifications (name, generation, manufacturer, instrument), method of measurement, applicability (failure/reliability criteria)*. Specifically for liver biopsy, indicate material used per center, i.e. percutaneous/transjugular/other, needle diameter.* | | p. 10-12 | |
| *8.5. Biological samples: description of method of collection, transport, storage*.* | | p-10-12 | |
| *8.6. Specify how the index tests were calculated.* | | p.10-13 | |
| *8.7. Specify how the risk for false negative/positive results was taken into account.* | | Fasting conditions; exclusion of patients  with severe alcoholic hepatitis  Multiple regression analyses of predictors  for MFAP4 | |
| *Specifically for liver biopsy:* | | | |
| *8.8. How sample bias was limited: minimal biopsy size (length)*, number of portal tracts required, number of fragments.* | | p.10  Biopsies were considered to be of adequate quality in the absence of cirrhosis if they were >10 mm length and contained >5 portal tracts. | |
| *8.9. Methods for histological assessment: human/automated reading*, local/central reading*, number and expertise of pathologists*, single/double reading*, consensus methods.* | | p.10-12  All evaluations were performed by one expert pathologists | |
| *8.10. Scoring system used (Metavir, Ishak, Scheuer, etc.).* | | p.10-12  Kleiner | |
| **9. Definition of and rationale for the units, cut-offs*, and/or categories of the results of the index tests and the reference standard.** | | p.15 | |
| **10. The number*, training and expertise* of the persons executing and reading the index tests and the reference standard.** | | p.10-12 | |
| **11. Whether or not the readers of the index tests and reference standard were blind (masked) to the results of the other test and describe any other clinical information available to the readers.** | | All investigations performed on the same  day. The results of MFAP4 were not available at  time of elastography and other noninvasive  tests. The pathologist was blinded to the results of all tests | |
| **12. State if the study is conducted on an intention-to-diagnose basis or if the analysis is per-protocol (i.e. with exclusion of failed/unreliable fibrosis test(s)/reference measurements).** |  | | p.17 and Table 2  We used both intention-to-diagnose and per-protocol analysis |
| *12.1. If intention-to-diagnose analysis, specify how failure and unreliable test(s)/reference are taken into account in the analysis. a* | | p.17 | |
| **13. Methods for calculating or comparing measures of diagnostic accuracy, and the statistical methods used to quantify uncertainty (e.g. 95% confidence intervals).** | | | |
| *Specify:* | | | |
| *13.1. Detailed sample size calculation.* | | Not performed | |
| *13.2. Statistical methods used to quantify uncertainty (e.g. 95% confidence intervals).* | | p.12-13  95% Confidence Interval was reported | |
| *13.3. Control of multiple comparisons that increases type I error: multiple comparisons of tests (e.g. Bonferroni correction, etc.), multiple diagnostic targets.* | | No multiple comparisons were performed | |
| *13.4. Method for calculation of fibrosis test(s) diagnostic cut-offs.* | | p.13 & p.16  Youden index. Rule-in and Rule-out cutoffs by setting sensitivity and specificity to 90% | |
| *13.5. Method for validation of new test(s) or new calculated diagnostic cut-off(s) (e.g. external validation set, internal validation by bootstrapping, etc.).* | | p.13  Patient cohort was split into a training and validation cohort depending on whether or not enrolled by July 31, 2014 | |
| *13.6. Method for control of center/operator effect.* | | p.9  Single center study | |
| *13.7. Method for control of spectrum effect if unrepresentative prevalence of fibrosis stages (e.g. Obuchowski index, DANA, etc.).* | | p.9 & Table 1.  We had representative prevalence of fibrosis during ALD in the training and validation cohort, as we recruited from both primary and secondary care | |
| *13.8. Method for control of misclassification errors by the reference test.* | | No method proposed, however we identified independent factors beyond fibrosis stage that could impact serum concentration of MFAP4 (AST) | |
| *13.9. Use of a reference without gold standard.* | | Not done | |
| *13.10. Analysis of discordances between reference/index test(s). b* | | p.16 | |

*.*

| **14. Methods for calculating test reproducibility.** | | Not performed | |
| --- | --- | --- | --- |
| **RESULTS** | | *Report:* | |
| Participants  **15. When study was performed, including beginning and end dates of recruitment.** |  | | p.13 |
| **16. Clinical and demographic characteristics of the study population (e.g. age*, sex*, spectrum of presenting symptoms, comorbidity, current treatments, recruitment centers).** | | Table 1 | |
| *16.1. For liver biopsy: size (length)*, number of portal tracts, number of fragments.* | | Table 1 | |
| *16.2. For index test(s): confounding factors that potentially influence the test(s) results (flare-up, inflammation, other liver lesions, intrinsic characteristics, etc.).* | | Appendix | |
| **17. The number of participants satisfying the criteria for inclusion who did or did not undergo the index tests and/or the reference standard*; describe why participants failed to undergo either test (a flow diagram is strongly recommended).** | | Appendix | |
| *17.1. If per-protocol analysis, report comparisons between patients excluded due to failed/unreliable test(s)/reference and patients with reliable fibrosis test(s)/reference.* | | p.17  ITD analysis | |
| Test results  **18. Time-interval* between the index tests and the reference standard, and any treatment administered between.** |  | | p.10  Same-day index and reference |
| **19. Distribution of severity of disease (define criteria) in those with the target condition*; other diagnoses in participants without the target condition.** | | Table 1 & Table 2 | |
| *19.1. Specify the prevalence* of the diagnostic condition (spectrum effect).* | | Table 2 | |
| **20. A cross tabulation of the results of the index tests (including indeterminate and missing results) by the results of the reference standard; for continuous results, the distribution of the test results by the results of the reference standard.** | | | |
| *20.1. Presentation of contingency tables, box/scatter plots.* | | Figure 1, Figure 3, Table 2 | |
| **21. Any adverse events from performing the index tests or the reference standard.** | | p.13  No adverse events due to index test. However two patients required intervention due to bleeding episode after performing the liver biopsy. | |
| Estimates  **22. Estimates of diagnostic accuracy* and measures of statistical uncertainty (e.g. 95% confidence intervals).** |  | | 95 % CI are performed for regression models and AUROC |
| *22.1. Specify sensitivity* and specificity* with 95% confidence intervals; ROC analysis.* | | Table 2 | |
| *22.2. Analyzing discordances between fibrosis tests(s)/reference. b* | | p.15-16 & Figure 4 | |
| **23. How indeterminate results, missing data and outliers of the index tests were handled.** | | | |
| *23.1. How missing/failure/unreliable results of index test(s)/reference were handled (intention-to-diagnose/per-protocol analysis). a* | | ITD & Per-protocol analysis | |
| *23.2. How outliers of the index tests were handled.* | | p.12  Outliers were included. | |
| **24. Estimates of variability of diagnostic accuracy between subgroups of participants, readers or centers, if done.** | | Not done  However we did perform multivariate analysis of factors influencing circulating MFAP4 beyond fibrosis. | |
| **25. Estimates of test reproducibility, if done.** | | Not done | |
| **26. Estimates of cost-benefit.** | | Not done | |
| **DISCUSSION**  **27. Discuss the clinical applicability of the study findings.** | |  | |
| *27.1. Discuss the representativeness of the study sample and recruiting centers (i.e. spectrum effect, etc.).* | | p.19-20 | |
| *27.2. Discuss the interpretation of fibrosis test(s) results in clinical practice.* | | p.19-20 | |
| *27.3. Discuss the clinical relevance of the study results.* | | p.19-20 | |

*a Items 12.1 and 23.1 are redundant but retained since they can be located in different paragraphs within an article*

*b Items 13.10 and 22.2 are redundant but retained since they can be located in different paragraphs within an article*

This file is the proprietary of AFEF and can be reproduced without authorization.

**Explanations**: see glossary

**Authors**: ARDENT group (see details in glossary) and AFEF (French Association for the Study of the Liver)

Version: February 2015

11. Figure : Relation between serum MFAP4 and nonalcoholic fatty liver disease activity (NAS) score


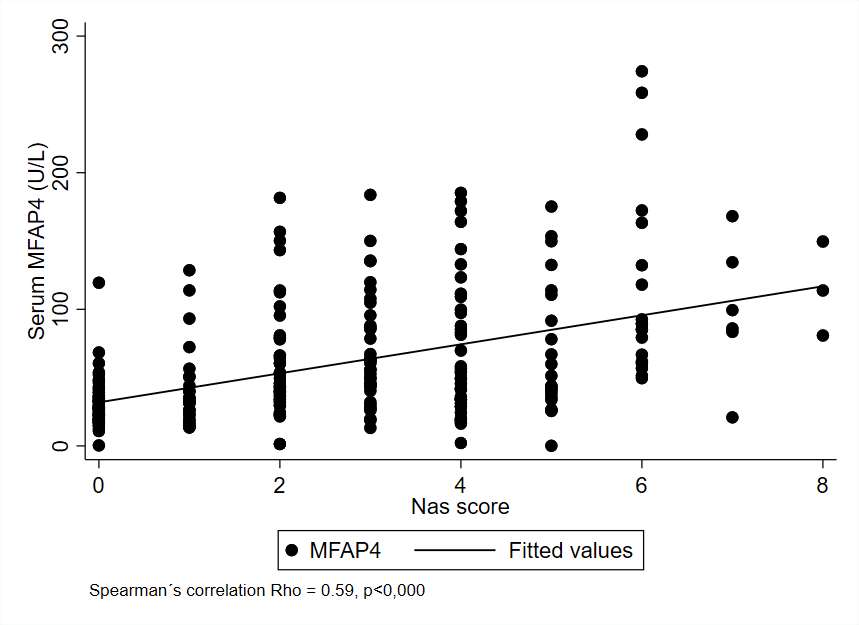


Scatterplot of serum MFAP4 and nonalcoholic fatty liver disease activity (NAS) score in 266 patients with prior or current alcohol overuse.

12. Figure: Serum MFAP4 according to collagen proportionate area


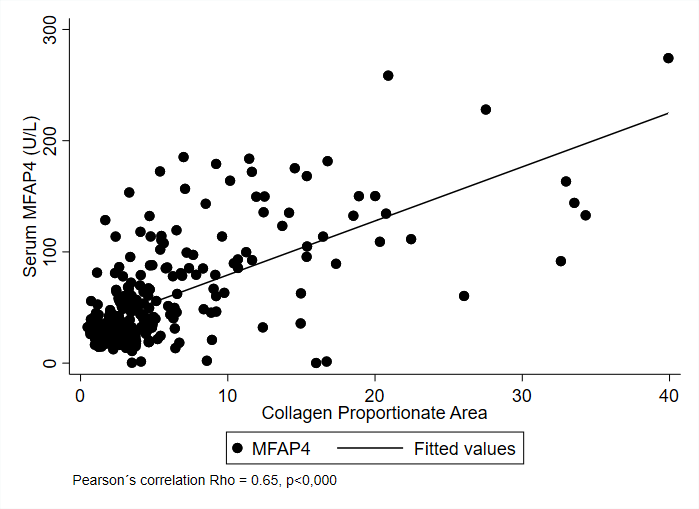


Scatterplot of serum levels of MFAP4 and collagen proportionate area in liver biopsies.
